# Soft Tissue Stiffness and Functional Knee Outcomes in Female Handball Players Following a Knee Injury: A Cross-Sectional Study

**DOI:** 10.3390/jcm15020891

**Published:** 2026-01-22

**Authors:** Joanna Mencel, Alicja Noculak, Tomasz Sipko

**Affiliations:** 1 Department of Physiotherapy in Motor Organ Dysfunction and Kinesiology, Faculty of Physiotherapy, Wroclaw University of Health and Sport Sciences, 51-612 Wrocław, Poland; 2 Department of Physiotherapy and Occupational Therapy, Faculty of Physiotherapy, Wroclaw University of Health and Sport Sciences, 51-612 Wrocław, Poland; alanoculakk@gmail.com (A.N.); tomasz.sipko@awf.wroc.pl (T.S.)

**Keywords:** patellar tendon, quadriceps muscle, Lysholm Knee Score

## Abstract

**Background/Objectives**: The aim of our study was to evaluate the transverse stiffness of selected soft tissues in the knee joint region on the previously injured and uninjured sides of female handball players and non-athlete women, in the lying and standing positions, and to investigate the relationship between stiffness, age, sporting practice, and clinical assessments of the knees. **Methods**: A total of 25 young female handball players (the SPORT group) and 27 healthy non-athletic individuals (the CONTROL group) were examined. The MyotonPRO device was used to measure the stiffness of the patellar tendon (PT), rectus femoris (RF), and biceps femoris (BF) muscles on both sides and in both positions. The function of the knee joints was clinically assessed using the Knee Outcome Survey—Sports Activities Scale and the Lysholm Knee Scoring Scale. **Results**: ANOVA indicated a significant effect of group (*p* < 0.003) on the PT’s stiffness, and a significant effect of position (*p* < 0.0001) on the PT, RF, and BF muscle stiffness. The SPORT group demonstrated significantly higher PT transverse stiffness when lying down (*p* < 0.01), but not when sitting up (*p* > 0.05), compared to the CONTROL group. Significant negative correlations were found between PT stiffness and both clinical scales in the SPORT group (rho from −0.39 to −0.71, *p* < 0.05). **Conclusions**: In female handball players, only the patellar tendon transverse stiffness was higher than in the control group. While this higher stiffness could indicate an adaptive rebuilding process, it was negatively correlated with the clinical assessment of joint function, meaning poorer knee joint function.

## 1. Introduction

Exercise leads to specific adaptive changes in the body, including those affecting the structure and mechanical properties of muscle-tendon units. The mechanical properties of skeletal muscles are crucial both for athletic performance and the risk of injury [1]. One such property is stiffness. Stiffness can be calculated by analyzing the damped natural oscillation resulting from short-term compression. This type of stiffness is referred to as transverse or dynamic stiffness. An accelerometer in a device called a Myoton detects these oscillations using a method called myotonometry. Numerous studies have confirmed the validity and reliability of stiffness assessments using the Myoton device [1,2,3], including those relating to the quadriceps femoris muscle and the patellar tendon (PT) [4], as well as the biceps femoris (BF) muscle [5]. The stiffness results obtained from selected lower limb muscles using the MyotonPRO device were highly reliable, particularly in standing and lying positions [6].

Given that motor behavior requires muscle-tendon units to generate force, an increasing number of authors recognize the need to study the mechanical properties of both muscles and tendons [7]. Tendons perform complex functions: they transmit force to the skeleton via collagen fibers and provide flexibility via elastin fibers. This enables them to store and release strain energy in a spring-like manner [8]. Therefore, to understand tendon function during training adaptation and optimize rehabilitation at pathological stages, if necessary, tendon stiffness must be assessed.

The tendons are highly responsive to increased mechanical loading, and they adapt by changing their mechanical, material, and morphological properties if the exercise intervention lasts longer than 12 weeks [9]. In addition to the tendon’s physiological adaptive responses to increased mechanical loading, which result in a functionally relevant improvement, excessive mechanical loading (overloading) is considered an important factor in the etiology of tendinopathy, which is characterized by activity-related pain, decreased strength, and reduced flexibility. However, there was no clear evidence that the mechanical or material properties of PT are altered in tendinopathy, while such changes were found in the Achilles tendon [10].

The PT was stiffer in weightlifting athletes than in non-athletes; however, both groups included men and women from 18 to 55 years old [11]. The elite footballers exhibited higher PT stiffness values than the control group, and it was suggested that PT stiffening may be an adaptive process that enhances force transmission and sports performance [12]. The authors found that female athletes in handball, volleyball, and hammer throwing had significantly higher Achilles tendon stiffness than female swimmers [13].

It should be noted that the majority of studies on muscle stiffness have been conducted on men [14,15]. This is an important issue, given that there are sex differences in myometric results, particularly in the lower limb muscles [5,16], though no differences have been observed between the dominant and non-dominant sides [17]. Age is another factor that affects the mechanical properties of soft tissues. Studies have shown that the rectus femoris muscle is stiffer in older people than in younger ones [3]. Other studies have evaluated the effects of maturation on myotonometric parameters and their role in predicting athletic performance in elite youth footballers [18]. Similarly, other authors have studied the effects of age and athletic training on the mechanical properties of the male quadriceps femoris muscle-tendon unit [19].

Handball has one of the highest injury rates (82.2%) of all Olympic sports [20]. This is due to the sport’s high physical demands, involving high-intensity activities such as running, jumping, landing, and changing direction. The findings suggest that knee injuries are a common issue among handball players [21]. Such injuries may lead to deficits and recurrences, so it is important to recognize and prevent them [22]. Regarding aspects related to injury prevention, information is limited, with the warm-up being the appropriate place to work on this aspect [20].

Injuries can range from mild to severe and may cause athletes to stop participating in sports activities temporarily or permanently. The causes of injuries are often complex. In team sports, risk factors include previous injuries, impaired proprioception, and musculoskeletal overload due to excessive training, fatigue during the second half of games, or the last hour of training [22]. Tissue stiffness, or the tissue’s ability to resist deformation, is also a factor. Interestingly, injuries can be caused by high or low stiffness values [23,24]. There is evidence that female athletes are more susceptible to injury than male athletes, particularly knee injuries [20,25]. An additional hour of training per week was associated with a higher risk of injury in female athletes. Studies have shown that athletes with a history of injuries are at high risk of overuse injuries, and the most common type of injuries found in the shoulders and knees of female athletes were overuse injuries with no any identifiable triggering event [22].

The stiffness of the soft tissues in the lower limbs of female handball players has not yet been compared with that of non-athlete women. Nor has the stiffness of these tissues been evaluated in female handball players with a history of knee injury. We chose to evaluate the mechanical properties in two positions (lying and standing), which will allow us to determine the stiffness values of the selected soft tissues in the lower limb in a relaxed state and when activated by the nervous system to maintain a habitual standing position. Although previous studies have focused on other skeletal muscles, they have demonstrated the potential of myotonometry for this purpose. For instance, authors have revealed that the lumbar muscle [26] and right and left lumbar erector spinae muscles are stiffer in the standing position than in the lying position, with no significant differences between the two sides [27].

The aim of our study therefore was: (i) to evaluate the transverse stiffness of selected soft tissues in the knee joint region on the previously injured and uninjured sides of female handball players and healthy non-athlete women, in both the lying and standing positions; and (ii) to investigate the relationship between transverse stiffness and other variables, such as clinical assessments, sporting practice, and age. We hypothesized that female handball players would exhibit greater soft tissue stiffness on both sides than non-athletic women. We also anticipated that stiffness would be higher when standing than when lying for both groups. Furthermore, we expected to find a correlation between transverse patellar tendon stiffness and knee function in handball players during sports participation and daily activities, as assessed by clinical scales.

## 2. Materials and Methods

### 2.1. Study Design

Due to the aim of the study, an observational study was conducted using a cross-sectional design. Two groups of participants were recruited: female handball players who had experienced a unilateral knee joint injury (the SPORT group) and a control group of healthy individuals (the CONTROL group). The sample size was determined based on the results of previous studies [13].

### 2.2. Participants

The study primarily targeted young, healthy female handball players who met the following inclusion criteria: active female handball athletes (participating in training sessions and competitions without any recent injuries), a history of one knee injury for at least six months prior, no surgical treatment for past injuries, and high-performance sports training for at least 5 years. We also recruited non-athletes to form an age-matched control group. Exclusion criteria for all participants included: BMI > 30 m/kg^2^ [28], taking medications that affect the musculoskeletal and central nervous systems, chronic disease, and a history of lower limb injury (for the CONTROL group), or acute/subacute injury (an injury within a period of less than six months; for the SPORT group). Ultimately, the study group (the SPORT group) comprised 25 female handball players, while the CONTROL group comprised 27. Because the subjects in the sports group had a history of unilateral injuries, we present the clinical characteristics related to this issue. The most common knee injuries were contusions (7), sprains (4), and anterior cruciate ligament (ACL) ruptures (4). The next most common injuries were patellar injuries and meniscus tears (3 and 2 women, respectively). Two athletes reported posterior cruciate ligament tears. The least common injuries were medial collateral ligament tears coexisting with meniscus tears (3). Anthropometric characteristics of the participants are shown in Table 1.

### 2.3. Measurements

All procedures were performed by one person, a physical therapist who was trained in the procedures being performed. These were conducted at similar times for everyone. Subjects in the sports group underwent measurements before training. The examinations were performed in the following order: an interview to detect inclusion and exclusion factors, an interview about sporting activities including competition seniority and training hours per week, as well as injuries, determination of body weight and height (Seca Deutschland, Seca 7, Hamburg, Germany), and a clinical assessment of the knee joints and myometric measurements. All participants gave their written informed consent before the study began. The study was conducted according to the Declaration of Helsinki, and the protocol was approved by the Ethics Committee of the Wroclaw University of Health and Sport Sciences (no 1/2025).

### 2.4. Clinical Assessment of the Knee Joints

The function of the knee joints was assessed using two standardized clinical scales: The Lysholm Knee Scoring Scale (LKSS) and the Knee Outcome Survey—Sports Activities Scale (KOS—SAS). An overall score is produced on a point scale from 0 to 100 by evaluating eight factors (such as limp, use of a cane or crutches, locking sensation in the knee, ability to climb stairs). A score of ‘excellent’ is given for 95–100 points, ‘good’ for 84–94 points, ‘sufficient’ for 65–83 points, and ‘insufficient’ for less than 65 points. The LKSS has been validated for use with patients presenting with a variety of knee injuries, including ACL and meniscal injuries [29,30,31].

The KOS—SAS is a patient-reported outcome measure that evaluates functional limitations due to knee disorders during sports activities. The result of the KOS—SAS is presented as a percentage, with higher values indicating better functional status. It was demonstrated that the KOS—SAS is a reliable and valid tool for assessing functional limitations of the knee [32]. In our study, we used the Polish versions of both questionnaires/scales. Both underwent cultural adaptation and validation, and were found to have similar levels of reliability and validity to the original language versions [33,34].

### 2.5. Mechanical Properties by Myometry—The Transverse Stiffness

The MyotonPRO device (Myoton AS, Tallinn, Estonia) was used to measure transverse stiffness in two positions: lying down and standing up in the habitual position (Myoton AS, Tallinn, Estonia). First, when the participant was lying relaxed, points for myometric measurements were determined by palpation and in accordance with previous recommendations [4,35] on selected soft tissues: the rectus femoris muscle (RF), the patellar tendon (PT), and the biceps femoris muscle (BF) of both lower extremities. The sites for measurements were as follows: for RF—two-thirds between the anterior superior iliac spine and the superior pole of the patella, for PT—midway between the distal part of the patella and the tibial tuberosity, and for the long head of the biceps femoris muscle—midway between the ischial tuberosity and the head of the fibula. We considered that only selecting the same measurement points would allow us to compare the results, as indicated by previous results [36]. Then, the testing end of the device was applied to the marked points, generating five discrete compressions (five compression modes; an initial force of 0.18 N, and a compression force of 0.4 N) that briefly deformed the tissue being tested. This resulted in damped natural oscillations, which were recorded by an accelerometer built into the device. Transverse stiffness was calculated as the ratio of the applied force to muscle deformation [N/m]. A previous study showed that the five compression mode is valid for assessing the mechanical properties of the lower limb myotendinous unit, specifically the RF and PT [37], which is why this number was used in our study. A request to relax the muscles preceded each measurement. As measurements were taken in two positions, after the lying position, participants assumed their habitual standing position, and measurements were taken at the same points in the same order. While a certain level of muscle activation is required to counteract the force of gravity in this position, the specific strategies used by the subjects to maintain it were not considered in the study.

### 2.6. Statistical Analysis

Data analysis was performed using the STATISTICA software package v. 13.3 (StatSoft Poland, Cracow, Poland). The Shapiro–Wilk test was used to assess the normality of the data distribution. An evaluation of possible differences in anthropometric characteristics and clinical scale scores was performed using a *t*-test for independent groups and a Mann–Whitney test, respectively. ANOVAs (separately for RF, BF, and PT) were used to determine the effects of group (SPORT/CONTROL), body position (lying/standing), and side (injured or right/non-injured or left) on the transverse stiffness. The results are presented as means with 95% confidence intervals (CIs). The effect size was determined using η^2^_p_. The Tukey test was used for post hoc analysis. Additionally, Spearman’s correlation test was used to examine the relationship between transverse stiffness and the clinical evaluation of the knee joints, and between transverse stiffness, age, and years of sports practice for RF, BF, and PT in both body positions. The strength of the correlation was interpreted according to the thresholds set out in [38]. The level of statistical significance was set at α ≤ 0.05, with Bonferroni correction applied to account for multiple comparisons in both the ANOVA and the correlation analysis.

## 3. Results

### 3.1. Anthropometric Characteristics and Clinical Assessments of Knee Joints

A *t*-test for independent groups was performed to compare the groups’ anthropometric parameters. Only the body mass variable differed significantly between the groups (t(50) = 2.07, *p* = 0.042). There were no significant differences in age, height, or BMI (*p* > 0.05; Table 1). The statistics confirmed the differences in sports participation between the two groups of women (*p* < 0.001, Table 1).

Mann–Whitney tests were performed on two clinical assessment scores to compare the results between groups. The results showed that the CONTROL group had significantly better knee joint function than the SPORT group (*p* < 0.0001). Results are presented in Table 2.

### 3.2. The Transverse Stiffness of the RF Muscle

The results of the ANOVA analysis showed that the group factor did not significantly impact the transverse stiffness of the RF muscle (F(1, 50) = 0.304, *p* = 0.58; η^2^_p_ ≈ 0.006. However, position F(1, 50) = 10.49, *p* = 0.002; η^2^_p_ ≈ 0.17 and side F(1, 50) = 23.85, *p* < 0.00001; η^2^_p_ ≈ 0.32 did.

There was also significant interaction between side x group (F(1, 50) = 7.75, *p* = 0.007; η^2^_p_ ≈ 0.13. The results of the ANOVAs are summarized in Table 3.

**Figure 1:**
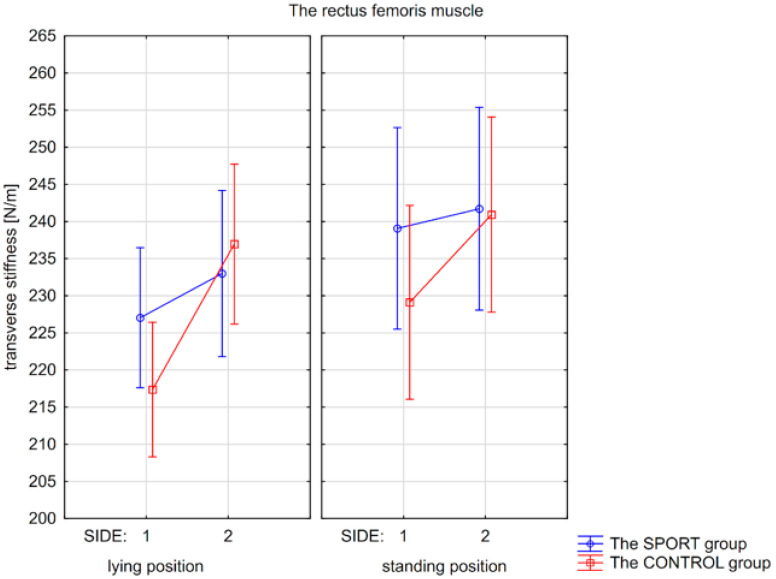
Mean values with confidence intervals of transverse stiffness of the rectus femoris muscle measured on the non-injured/or right (side 1) and the injured/or left side (side 2) in the lying and standing positions for the SPORT and the CONTROL groups.

Post hoc tests revealed higher transverse stiffness of the RF muscle in the standing position than in the lying position for the non-injured side (*p* < 0.002) for the SPORT group and for the right side for the CONTROL group (*p* < 0.002). For the CONTROL group, there was also significantly higher stiffness in the left RF muscle compared to the right RF muscle in the lying position (*p* < 0.0002) and standing position (*p* < 0.002; Figure 1).

### 3.3. The Transverse Stiffness of the BF Muscle

The ANOVA results indicated a significant effect of body position F(1, 50) = 25.57, *p* < 0.001, η^2^_p_ ≈ 0.33, and side, F(1, 50) = 5.65, *p* = 0.021, η^2^_p_ ≈ 0.10, on the transverse stiffness of BF, but not group factor, F(1, 50) = 0.25, *p* < 0.61, η^2^_p_ ≈ 0.004. For more details, see Table 3.

**Figure 2:**
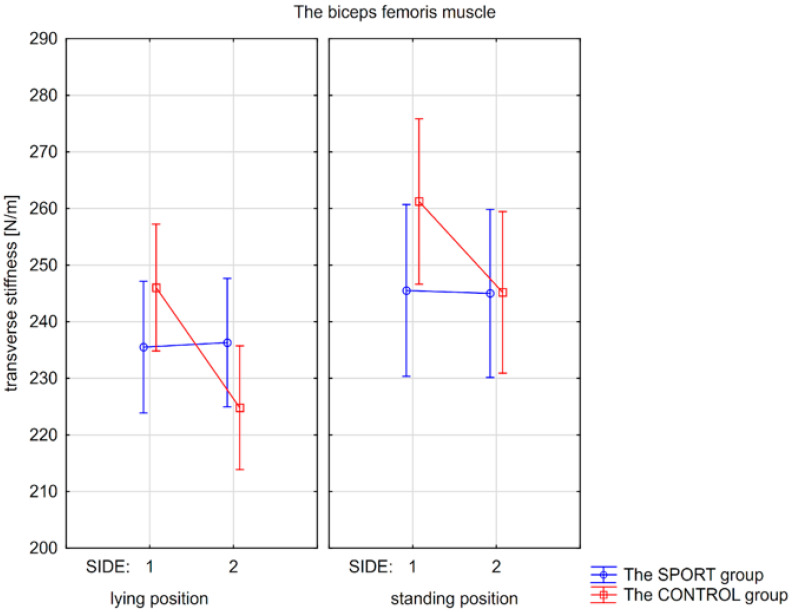
Mean values with confidence intervals of transverse stiffness of the biceps femoris muscle measured on the non-injured/or right (side 1) and the injured/or left side (side 2) in the lying and standing positions for the SPORT and the CONTROL groups.

Post hoc tests showed that significant differences were only found in the CONTROL group, but not in the SPORT group. Significantly higher stiffness in standing compared to the lying position was found for the left BF (*p* = 0.006), and higher stiffness of the right BF compared to the left BF in the lying position (*p* = 0.004; Figure 2).

### 3.4. The Transverse Stiffness of the PT

Results of ANOVA indicated a significant effect of group F(1, 50) = 15.64, *p* < 0.003; η^2^_p_ ≈ 0.23 and position F(1, 50) = 39.11, *p* < 0.0001; η^2^_p_ ≈ 0.43 on the PT’s transverse stiffness. There were also significant effects of interaction between position x group F(1, 50) = 8.34, *p* < 0.006; η^2^_p_ ≈ 0.14. The details are shown in Table 3.

Post hoc tests indicated significantly higher transverse stiffness of the PT in the SPORT group than in the CONTROL group, in both the non-injured/right (*p* < 0.01) and injured/left sides (*p* < 0.01) in the lying position, but not in the standing position (*p* > 0.05).

**Figure 3:**
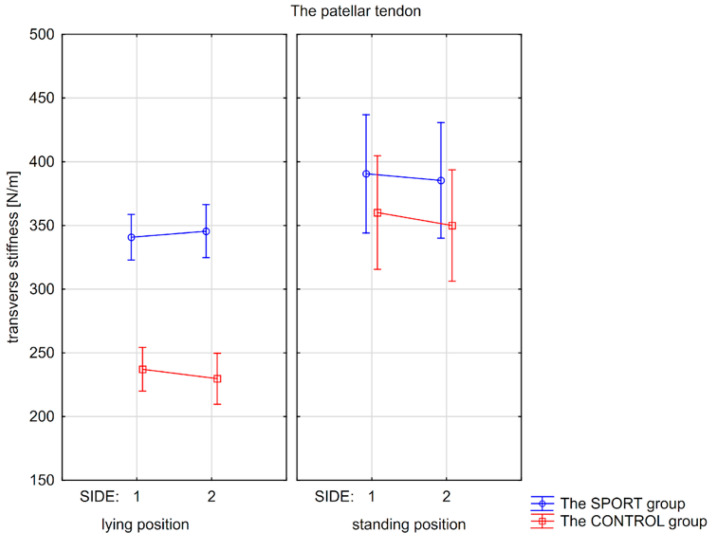
Mean values with confidence intervals of transverse stiffness of the patellar tendon measured on the non-injured/or the right (side 1) and injured/or the left side (side 2) in the lying and standing positions for the SPORT and the CONTROL groups.

In the SPORT group, significantly higher stiffness of PT was found in the standing position compared to the lying position for non-injured and injured sides (*p* < 0.001 and *p* < 0.01, respectively). Similarly, significantly higher PT stiffness was revealed for both sides in the standing position compared to the lying position in the CONTROL group (*p* < 0.001; Figure 3).

### 3.5. The Correlation Analysis—The SPORT Group

The results of the Spearman test indicated significant, moderate, and positive correlations between the transverse stiffness of the PT and age in the lying position for both lower limbs (rho = 0.44 and 0.46, respectively, *p* < 0.05), as well as a large correlation in the standing position (rho = 0.589 and 0.527, respectively, *p* < 0.05) in the SPORT group. A similar result was observed for the correlation between PT stiffness and sports practice (rho 0.432–0.598, *p* < 0.05; Table 4). Significant negative correlations of moderate to high strength were also noted between PT stiffness in the standing position and both clinical scales in the SPORT group (*p* < 0.05). In this group, significant negative medium correlations were also observed between PT stiffness in the non-injured limb in lying and the LKSS scale (*p* < 0.05).

There was no significant correlation between the transverse stiffness of the BF muscle and the variables of the anthropometric, sports, and clinical scales (*p* > 0.05) in the SPORT group. There was also a significant, moderate positive correlation between the stiffness of the RF muscle and height in the SPORT group (*p* < 0.05). Detailed results of the correlation analysis are shown in Table 4.

### 3.6. The Correlation Analysis—The CONTROL Group

The results of the Spearman test indicated significant, negative, moderate correlation between transverse stiffness of the left RF muscle in lying position and the height (*p* < 0.05) in the CONTROL group (Table 5). Significant, negative correlations of moderate to large strength were also noted between the stiffness of RF muscle in both the lying and standing positions with clinical scales (*p* < 0.05), except for the right RF muscle in the lying position and KOS—SAS (*p* > 0.05; Table 5).

## 4. Discussion

Our study examined the transverse stiffness of selected soft tissues in the knee joint region on the previously injured and uninjured sides of female handball players and healthy, non-athletic women. The study also explored the relationship between stiffness and clinical assessments of lower limb function, sporting practice, and anthropometric characteristics.

One of the most important findings was that the transverse stiffness of the patellar tendon was significantly higher in female handball players than in the control group when lying down. This could be a result of adaptation to training. There were no differences between the groups in terms of the transverse stiffness of the antagonist thigh muscles. Furthermore, significant relationships were identified between the transverse stiffness of the patellar tendon, age, and years of sports experience, as well as two clinical assessment results, in both positions and on both sides of the handball players. Interestingly, increased stiffness of the PT was linked to lower scores on both clinical evaluation scales in this group. These scores were significantly worse than those of the healthy, non-training control group. We are unable to determine the cause of the increased stiffness of the PT at rest in female handball players compared to healthy non-athletic women. However, the results emphasize the importance of proper training load management to prevent functional limitations related to increased tendon stiffness.

In contrast, in the control group, only RF stiffness was associated with both clinical scales, on which the subjects scored an average of 97%. In other words, RF stiffness is associated with normal knee joint function in healthy, non-athletic women.

### 4.1. The Group Difference in the Transverse Stiffness of the PT

Our results showed that the transverse stiffness of the PT alone was higher in handball players (340 ± 47 and 345 ± 45 N/m for the non-injured and injured sides, respectively) than in the control group (237 ± 41 and 229 ± 56 N/m for the left and the right sides, respectively) when measured in a relaxed, supine position. Interestingly, no differences were observed in the standing position for PT, nor between the muscles (RF and BF) in the lying and standing positions. On the one hand, similar to our results for the control group, values of PT transverse stiffness at rest were obtained in studies conducted on healthy young participants (220 and 223 N/m for the dominant and non-dominant lower limbs, respectively, age 24.7 ± 1.6), which could be considered normative values [4]. On the other hand, previous research found similar results regarding higher PT stiffness in the training group of weightlifting athletes compared to the non-training group [11] while the participants were lying down with their legs extended. However, the values they obtained for both groups (327.30 ± 93.19 N/m and 319.82 ± 84.16 N/m for the sports and control groups, respectively) were similar to ours for the sports group. In our opinion, this is related to the participants’ diverse age range (18–55 years old), as other studies have found that PT stiffness increases with age [39]. At the same time, it is worth noting that studies have shown that myometry can detect differences in Achilles tendon stiffness in different sports, such as comparing swimmers with handball, volleyball, and hammer throw athletes [40], indicating a limited ability to compare results between athletes of different sports. From this perspective, our results can serve as an important reference point for others. The increased PT stiffness in our sport group may indicate an adaptation to training, as presented by the model, which predicts changes in tendon mechanical properties in response to loading [41], but we are unable to determine the actual causes of the observed results.

Our correlation results also indicated that PT stiffness increases with years of sporting practice (and age) when standing among female handball players. However, we did not observe such a relationship in the control group. We demonstrated that an increase in the handball players’ PT transverse stiffness was associated with a deterioration in knee joint function, as assessed by two clinical scales, particularly when standing. It is important to note that this negative correlation may be mediated by other factors, such as psychological factors, the severity of past injuries, and the history of pain, which were not assessed in the study.

Previous study has shown that lower limb stiffness has an impact on both performance and injury risk [42]. In terms of performance, greater stiffness is associated with greater speed and jump height. The authors of other studies have shown that an increase in tendon stiffness (calculated from the slope of the force-elongation curve) acts as a protective mechanism for tendon tissue integrity, and due to the mechanical load profile, musculoskeletal imbalances are particularly concerning for athletes practicing jumping disciplines, in which tendinopathy is very common [43]. Previous studies indicate that in the context of tendinopathy, imbalances between muscle strength and tendon stiffness may be of particular importance [43,44]. In a longitudinal study by other authors, maximum patellar tendon tension was used to identify muscle–tendon imbalances and recommend personalized exercises as a remedy [44]. Their approach was effective in promoting uniform adjustment of knee extensor muscle strength and patellar tendon stiffness, as well as in reducing the incidence of muscle–tendon imbalance in adolescent female athletes. This concept can be used to prevent tendon injuries caused by overload, especially in populations with a high prevalence of tendinopathy and muscle–tendon imbalance [44]. Although tendinopathy was not diagnosed in any of the study participants, an imbalance between the stiffness of the rectus femoris muscle and the tendon can be inferred.

The results of our study emphasize that excessively high stiffness of the PT may also be associated with functional limitations. It therefore seems that there is an ‘optimal’ level of stiffness. Since sports training modifies stiffness both locally (as assessed by Myoton) and globally (as mentioned before in terms of the lower limb stiffness), the optimal solution would be to determine the threshold value above which soft tissue function deteriorates and the likelihood of injury increases. As other authors have pointed out, a well-designed, periodic training program should positively impact the stiffness of athletes’ soft tissues [45]. From our perspective, the Myoton device is considered to be an effective tool for objectively monitoring changes in the stiffness of muscle-tendon units in response to training. Accurately predicting ligament fatigue failure under repetitive loading is crucial for improving injury prevention and maintaining the musculoskeletal health of athletes. The pathogenesis of musculoskeletal disorders is closely associated with the cumulative damage and fatigue failure behavior of fibrous connective tissues under long-term repetitive loading. Recently, a multimodal intelligent assessment framework for ligament fatigue life was developed to address critical gaps in conventional approaches to nonlinear biomechanical modeling and real-time fatigue failure assessment [46]. The authors of the study found that differences in the function and structure of the ACL, PT, and lateral ankle ligaments directly impact the distribution of mechanical stress and the fatigue response under identical load patterns.

### 4.2. The Transverse Stiffness of Soft Tissues in the Knee Region in the Lying vs. Standing Position

Our study showed that body position affects the transverse stiffness of the examined soft tissues. Our assumption that stiffness values are higher in the standing position than in the lying position was confirmed for PTs (in both lower limbs), the RF muscle (in one lower limb) in two groups, and the BF muscle (in the control group only). For the non-injured limb of female handball players, the RF stiffness increased from 227 N/m in the lying position to 239 N/m in the standing position (an increase of ±5%), and for the right RF in healthy individuals, it increased from 225 N/m to 237 N/m (a similar increase of 5%). The increase in stiffness in standing compared to lying down for the injured and left side, respectively, was about 3% and did not reach statistical significance. No significantly higher levels of BF muscle stiffness were found in the SPORT group while standing (the difference was approximately 4% and 3% for the non-injured and injured sides, respectively), whereas in the control group, they were found for the left BF only (increase in stiffness when standing by approx. 9%, from 224 N/m to 245 N/m). Currently, there are no studies available that address the effect of changing position on the stiffness of the soft tissue we selected for our study. Therefore, they cannot be referred to. Previous studies have shown that as the target force level increases, the muscle stiffness value assessed by myotonometry increases [47]. Therefore, it is reasonable to assume that maintaining a standing position requires a certain level of muscle activation from the central nervous system, i.e., an increase in force generation and, consequently, an increase in muscle transverse stiffness. In our study, it was evident for PT (on both sides and in both groups), but not for the examined muscles, which may be related to transcutaneous myometric measurements and skinfold thickness. Earlier studies have shown that the thickness of skinfold is important for myometric measurements in individuals, even with a normal BMI [5], particularly with regard to the muscles in women’s lower limbs due to the distribution of adipose tissue [16]. For PT measurements, however, this fold is smaller, meaning that it may have had the least impact on the results. This may explain the significant increase in PT stiffness values when standing compared to lying down (by approximately 50% in the control group and approximately 11% and 15% in the sports group due to higher stiffness values in the lying position).

### 4.3. The Differences in the Muscle Stiffness Between the Sides in the CONTROL Group

Based on the available information [17,48], we did not assume any differences in stiffness between the right and left sides of the examined soft tissues in the control group. However, due to unilateral injuries in the sport group, we included this factor in the analysis, despite there being a lack of literature on this topic. Interestingly, this side factor had a significant effect on muscle stiffness, but not on PT stiffness. A detailed analysis showed that the differences between the sides were significant only in the control group, not in the sport group (the non-significant difference was ~1%). In the control group, significant differences in muscle stiffness between the right and left sides ranged from 4% to 8%, with greater differences observed at rest. Our results contradict those of other authors who have shown no difference in stiffness between the right and left rectus femoris (RF) muscles in healthy women [23]. However, the subjects in their study were older than those in ours (29.92 ± 9.63). Other researchers have also found no differences in PT and RF stiffness between the dominant and non-dominant lower limbs [4], but the study included both women and men, which may have affected the results. Based on the results obtained from both our groups, further investigation of the side factor is necessary to reach a clear conclusion.

### 4.4. Practical Implications

It can be assumed that there is a threshold value for the transverse stiffness of PT. Exceeding this threshold will result in a deterioration in knee joint function, as assessed using clinical scales. It seems important to determine the threshold, but this requires further research involving a larger number of women. While we cannot identify the exact reasons for our results, it seems reasonable to recommend regular, objective assessments of muscle-tendon stiffness in female athletes. Our results also emphasize the need for further injury prevention strategies in women’s handball.

### 4.5. Limitations of the Study and Future Direction

Some limitations should be pointed out. Firstly, based on the cross-sectional study design, we cannot make causal claims about the deterioration of knees in handball players. Secondly, different musculoskeletal injuries may affect the studied parameters in different ways, and a detailed analysis of individual injuries could provide a better understanding of the results.

Furthermore, as only women participated in our study, the conclusions should not be applied to men. Furthermore, as only women participated in our study, this means that the conclusions should not be extrapolated to men. However, the fact that the study was conducted only on women was intentional in order to eliminate sex-related differences in transverse stiffness, as well as due to the heterogeneous adaptation mechanisms to sports and injury in men and women [49]. The measurements were taken regardless of the phase of the menstrual cycle. Future studies should investigate this issue, particularly concerning the standing position, given that studies by other authors [50] have shown that the stiffness of certain lower limb muscles differs between the follicular phase and ovulation when measured during contraction rather than at rest. Additionally, electromyography was not used in our study to determine the muscle activation state. Therefore, it cannot be assumed that the measurements taken in the lying position were carried out in complete relaxation.

Nevertheless, the research highlights an interesting and important issue and should continue in order to eliminate limitations as much as possible and clearly identify the mechanism behind increased PT stiffness in female handball players.

## 5. Conclusions

Female handball players with a history of one knee injury had significantly higher patellar tendon stiffness in the lying position than matched non-athlete women. Additionally, the high PT stiffness values (but not RF and BF muscle values) recorded in both groups while standing showed a significant positive correlation with sports practice, which may indicate tendon adaptation to loads. High PT stiffness values are associated with deteriorating knee joint function in female handball players.

## Figures and Tables

**Table 1:** The anthropometric and sports characteristics of the females in the SPORT group (n = 25) and the CONTROL group (n = 27), with the results of the between-group comparison. Significant results are marked in bold.

Variable		The SPORT Group					The CONTROL Group				
	M	SD	Me	Min.	Max.	M	SD	Me	Min.	Max.	*p* Value
Age [years]	20	3.82	19	17	29	22	2.10	22	19	31	0.118
Height [m]	1.69	0.03	1.69	1.6	1.79	1.68	0.05	1.69	1.57	1.78	0.309
Body mass [kg]	63	6.03	63	54	75	59	6.08	60	49	70	**0.042**
BMI [kg/m^2^]	22	1.78	21	19	25	21	2.41	21	16	25	0.171
Sport practice [years]	10.16	2.74	9	6	15	1.07	0.93	1.5	0	2	**<0.0001**
Training hours per week	8.24	1.71	8	6.5	12	2.89	2.92	3	0	11	**<0.0001**

Abbreviations: M—mean value; SD—standard deviation; Me—median value.

**Table 2:** The result of the between-group comparison concerning two clinical scales of knee joint function in female handball players (the SPORT group) and the CONTROL group. Significant results are marked in bold.

Scale	The SPORT Group					The CONTROL Group					Between-Group Comparison	
	M	SD	Me	Min.	Max.	M	SD	Me	Min.	Max.	Z-Value	*p*-Value
LKSS	78.80	10.87	80	46	94	97.03	7.59	95	74	100	−4.89	**<0.0001**
KOS—SAS	84.66	9.16	85.5	52.7	92.7	97.03	3.49	98	87	100	−5.78	**<0.0001**

Abbreviations: M—mean value; SD—standard deviation; Me—median value; LKSS—Lysholm Knee Scoring Scale; KOS—SAS—Knee Outcome Survey—Sports Activities Scale.

**Table 3:** The results of three ANOVAs for the transverse stiffness of the rectus femoris (RF), the biceps femoris (BF) muscles, and the patellar tendon (PT). Significant results are marked in bold, with the adjusted α level taken into account for the number of comparisons made (0.025 for two, 0.0125 for four and 0.008 for six).

Transverse Stiffness	Factor	*F* Value	*p* Value	η^2^_p_	Observed Power
RF	Group	F(1, 50) = 0.30	0.583	0.006	0.08
	Position	F(1, 50) = 10.49	**0.002**	0.173	0.88
	Side	F(1, 50) = 23.85	**0.00001**	0.323	0.99
	position × group	F(1, 50) = 0.20	0.655	0.004	0.07
	side × group	F(1, 50) = 7.75	**0.007**	0.134	0.77
	position × side	F(1, 50) = 4.16	0.046	0.076	0.51
	position × side × group	F(1, 50) = 0.67	0.415	0.013	0.12
BF	Group	F(1, 50) = 0.25	0.619	0.004	0.07
	Position	F(1, 50) = 25.57	**0.000006**	0.338	0.99
	Side	F(1, 50) = 5.65	**0.021**	0.101	0.64
	position × group	F(1, 50) = 2.48	0.121	0.047	0.33
	side × group	F(1, 50) = 5.82	0.019	0.104	0.65
	position × side	F(1, 50) = 0.12	0.722	0.002	0.06
	position × side × group	F(1, 50) = 0.36	0.548	0.007	0.09
PT	Group	F(1, 50) = 15.63	**0.0002**	0.238	0.97
	Position	F(1, 50) = 39.11	**0.000001**	0.438	0.99
	Side	F(1, 50) = 0.32	0.569	0.006	0.08
	position × group	F(1, 50) = 8.34	**0.005**	0.143	0.80
	side × group	F(1, 50) = 0.29	0.589	0.005	0.08
	position × side	F(1, 50) = 0.35	0.554	0.007	0.08
	position × side × group	F(1, 50) = 0.11	0.737	0.002	0.06

**Table 4:** Results of Spearman’s rho correlations between transverse stiffness of the rectus femoris (RF) and biceps femoris (BF) muscles, and the patellar tendon (PT) for non-injured and injured lower limbs, and two body positions (lying and standing), as well as anthropometric, sports, and two clinical scales for the SPORT group. Significant results are marked with an asterisk and in bold. The adjusted α level was taken into account for the number of comparisons made (0.025 for clinical scales and 0.0125 for others).

THE SPORT GROUP								
Variable			Anthropometric and Sports				Clinical Scales	
Transverse Stiffness			Age	Height	Body Mass	Sport Practice	LKSS	KOS—SAS
RF	Non-injured	lying	0.064	0.267	−0.207948	0.083	−0.020	0.029
	Injured		0.251	0.159	−0.179873	0.263	−0.152	−0.113
	Non-injured	stand.	0.202	0.446 *	0.100347	0.256	−0.143	0.028
	Injured		0.291	0.414 *	0.182713	0.365	−0.041	0.074
BF	Non-injured	lying	−0.090	0.244	−0.060559	−0.110	0.197	0.130
	Injured		−0.057	−0.058	−0.263686	−0.112	0.244	0.254
	Non-injured	stand.	−0.204	0.164	−0.190899	−0.197	0.138	−0.013
	Injured		0.018	0.066	−0.253424	0.023	0.320	0.190
PT	Non-injured	lying	0.448 *	0.202	0.051658	0.482 *	−0.428 *	−0.337
	Injured		0.464 *	0.329	0.085246	0.432 *	−0.290	−0.251
	Non-injured	stand.	0.589 *	0.117	0.007520	0.598 *	−0.715 *	−0.571 *
	Injured		0.527 *	0.007	−0.215773	0.556 *	−0.396 *	−0.501 *

**Table 5:** Results of Spearman’s rho correlations between transverse stiffness of the rectus femoris (RF) and biceps femoris (BF) muscles, and the patellar tendon (PT) for the right and the left lower limb, and two body positions (lying and standing), as well as anthropometric, sports, and two clinical scales for the CONTROL group. Significant results are marked with an asterisk and in bold. The adjusted α level is taken into account for the number of comparisons made (0.025 for clinical scales and 0.0125 for others).

THE CONTROL GROUP								
Variable			Anthropometric And Sport				Clinical Scales	
Transverse Stiffness			Age	Height	Body Mass	Sport Practice	LKSS	KOS—SAS
RF	The right	lying	0.147	−0.223	0.036	0.091	−0.446 *	−0.372
	The left		−0.078	−0.418 *	0.113	0.238	−0.594 *	−0.630 *
	The right	stand.	−0.141	−0.039	0.033	0.221	−0.485 *	−0.410 *
	The left		−0.202	−0.349	0.129	0.306	−0.475 *	−0.464 *
BF	The right	lying	0.050	0.053	0.354	0.224	−0.181	−0.110
	The left		0.003	0.029	0.041	0.493 *	−0.304	−0.249
	The right	stand.	−0.120	−0.037	0.230	0.090	−0.091	−0.107
	The left		−0.110	−0.039	0.068	0.193	−0.302	−0.263
PT	The right	lying	0.146	0.013	0.125	0.170	−0.315	−0.362
	The left		0.160	−0.174	0.054	0.294	−0.230	−0.162
	The right	stand.	−0.060	−0.198	0.348	0.452 *	−0.087	−0.159
	The left		−0.245	−0.006	0.218	0.473 *	0.003	0.049

## Data Availability

The data that support the findings of this study are available on request from the corresponding author.

## Abbreviations

The following abbreviations are used in this manuscript:

ACL	Anterior cruciate ligament
LKSS	The Lysholm Knee Scoring Scale
KOS—SAS	The Knee Outcome Survey—Sports Activities Scale
RF	Rectus femoris muscle
BF	Biceps femoris muscle
PT	Patellar tendon

## References

[1] Lettner J., Królikowska A., Ramadanov N., Oleksy Ł., Hakam H.T., Becker R., Prill R. (2024). Evaluating the Reliability of MyotonPro in Assessing Muscle Properties: A Systematic Review of Diagnostic Test Accuracy. Medicina.

[2] Aird L., Samuel D., Stokes M. (2012). Quadriceps muscle tone, elasticity, and stiffness in older males: Reliability and symmetry using the MyotonPRO. Arch. Gerontol. Geriatr..

[3] Agyapong-Badu S., Warner M., Samuel D., Stokes M. (2016). Measurement of ageing effects on muscle tone and mechanical properties of rectus femoris and biceps brachii in healthy males and females using a novel hand-held myometric device. Arch. Gerontol. Geriatr..

[4] Chen G., Wu J., Chen G., Lu Y., Ren W., Xu W., Xu X., Wu Z., Guan Y., Zheng Y. (2019). Reliability of a portable device for quantifying tone and stiffness of quadriceps femoris and patellar tendon at different knee flexion angles. PLoS ONE.

[5] Lee Y., Kim M., Lee H. (2021). The Measurement of Stiffness for Major Muscles with Shear Wave Elastography and Myoton: A Quantitative Analysis Study. Diagnostics.

[6] Pruyn E.C., Watsford M.L., Murphy A.J. (2016). Validity and reliability of three methods of stiffness assessment. J. Sport Health Sci..

[7] Reiner M., Tilp M., Nakamura M., Konrad A. (2024). Is muscle stiffness a determinant for range of motion in the leg muscles?. Biol. Sport.

[8] Wiesinger H.P., Rieder F., Kösters A., Müller E., Seynnes O.R. (2016). Are Sport-Specific Profiles of Tendon Stiffness and Cross-Sectional Area Determined by Structural or Functional Integrity?. PLoS ONE.

[9] Bohm S., Mersmann F., Arampatzis A. (2015). Human tendon adaptation in response to mechanical loading: A systematic review and meta-analysis of exercise intervention studies on healthy adults. Sports Med.-Open.

[10] Obst S.J., Heales L.J., Schrader B.L., Davis S.A., Dodd K.A., Holzberger C.J., Beavis L.B., Barrett R.S. (2018). Are the Mechanical or Material Properties of the Achilles and Patellar Tendons Altered in Tendinopathy? A Systematic Review with Meta-analysis. Sports Med..

[11] Szajkowski S., Pasek J., Dwornik M., Cieślar G. (2024). Mechanical properties of the patellar tendon in weightlifting athletes—The utility of myotonometry. Adaptations of patellar tendon to mechanical loading. Acta Bioeng. Biomech..

[12] Cristi-Sánchez I., Danes-Daetz C., Neira A., Ferrada W., Yáñez Díaz R., Silvestre Aguirre R. (2019). Patellar and Achilles Tendon Stiffness in Elite Soccer Players Assessed Using Myotonometric Measurements. Sports Health A Multidiscip. Approach.

[13] Römer C., Czupajllo J., Wolfarth B., Sichting F., Legerlotz K. (2024). The Myometric Assessment of Achilles Tendon and Soleus Muscle Stiffness before and after a Standardized Exercise Test in Elite Female Volleyball and Handball Athletes—A Quasi-Experimental Study. J. Clin. Med..

[14] Klich S., Michalik K., Pietraszewski B., Hansen E.A., Madeleine P., Kawczyński A. (2024). Effect of applied cadence in repeated sprint cycling on muscle characteristics. Eur. J. Appl. Physiol..

[15] Pożarowszczyk B., Gołaś A., Chen A., Zając A., Kawczyński A. (2018). The Impact of Post Activation Potentiation on Achilles Tendon Stiffness, Elasticity and Thickness among Basketball Players. Sports.

[16] Mencel J., Jaskólska A., Marusiak J., Kisiel-Sajewicz K., Siemiatycka M., Kaminski L., Jaskólski A. (2021). Effect of gender, muscle type and skinfold thickness on myometric parameters in young people. PeerJ.

[17] Ramazanoğlu E., Usgu S., Yakut Y. (2020). Assessment of the mechanical characteristics of the lower extremity muscles with myotonometric measurements in healthy individuals. Physiother. Q..

[18] García-Santamaría A., Abelairas-Gómez C., Carrera S., Padrón-Cabo A., Rey E. (2024). Effects of maturation on myotonometric parameters and their predictors of athletic performance in elite youth soccer players. Sci. Rep..

[19] Charcharis G., Mersmann F., Bohm S., Arampatzis A. (2019). Morphological and Mechanical Properties of the Quadriceps Femoris Muscle-Tendon Unit From Adolescence to Adulthood: Effects of Age and Athletic Training. Front. Physiol..

[20] Vila H., Barreiro A., Ayán C., Antúnez A., Ferragut C. (2022). The Most Common Handball Injuries: A Systematic Review. Int. J. Environ. Res. Public Health.

[21] Bere T., Alonso J.M., Wangensteen A., Bakken A., Eirale C., Dijkstra H.P., Ahmed H., Bahr R., Popovic N. (2015). Injury and illness surveillance during the 24th Men’s Handball World Championship 2015 in Qatar. Br. J. Sports Med..

[22] Giroto N., Hespanhol Junior L.C., Gomes M.R., Lopes A.D. (2017). Incidence and risk factors of injuries in Brazilian elite handball players: A prospective cohort study. J. Med. Sci. Sports.

[23] Bradshaw E.J., Hume P.A. (2012). Biomechanical approaches to identify and quantify injury mechanisms and risk factors in women’s artistic gymnastics. Sports Biomech..

[24] Watsford M.L., Murphy A.J., McLachlan K.A., Bryant A.L., Cameron M.L., Crossley K.M., Makdissi M. (2010). A prospective study of the relationship between lower body stiffness and hamstring injury in professional Australian rules footballers. Am. J. Sports Med..

[25] Hoog P., Warren M., Smith C.A., Chimera N.J. (2016). Functional hop tests and tuck jump assessment scores between female division i collegiate athletes participating in high versus low ACL injury prone sports: A cross sectional analysis. Int. J. Sports Phys. Ther..

[26] Li Y., Yu J., Zhang J., Zhang Z., Wang X. (2022). Quantifying the stiffness of lumbar erector spinae during different positions among participants with chronic low back pain. PLoS ONE.

[27] Sipko T., Barczyk-Pawelec K., Piksa M., Mencel J. (2024). Impact of Standing and Sitting Postures on Spinal Curvature and Muscle Mechanical Properties in Young Women: A Photogrammetric and MyotonPro Analysis. Med. Sci. Monit..

[28] Gapeyeva H., Vain A. (2008). Methodical Guide: Principles of Applying Myoton in Physical Medicine and Rehabilitation.

[29] Briggs K.K., Lysholm J., Tegner Y., Rodkey W.G., Kocher M.S., Steadman J.R. (2009). The reliability, validity, and responsiveness of the Lysholm score and Tegner activity scale for anterior cruciate ligament injuries of the knee: 25 years later. Am. J. Sports Med..

[30] Kocher M.S., Steadman J.R., Briggs K.K., Sterett W.I., Hawkins R.J. (2004). Reliability, validity, and responsiveness of the Lysholm knee scale for various chondral disorders of the knee. J. Bone Jt. Surg..

[31] Wang D., Jones M.H., Khair M.M., Miniaci A. (2010). Patient-reported outcome measures for the knee. J. Knee Surg..

[32] Irrgang J.J., Snyder-Mackler L., Wainner R.S., Fu F.H., Harner C.D. (1998). Development of a patient-reported measure of function of the knee. J. Bone Jt. Surg..

[33] Piontek T., Ciemniewska-Gorzela K., Naczk J., Cichy K., Szulc A. (2012). Linguistic and cultural adaptation into Polish of the IKDC 2000 subjective knee evaluation form and the Lysholm scale. Pol. Orthop. Traumatol..

[34] Szczepanik M., Jabłoński J., Bejer A., Bazarnik-Mucha K., Majewska J., Snela S., Szymczyk D. (2023). Validation of the Polish Version of Knee Outcome Survey Activities of the Daily Living Scale in a Group of Patients after Arthroscopic Anterior Cruciate Ligament Reconstruction. J. Clin. Med..

[35] Mullix J., Warner M., Stokes M. (2012). Testing muscle tone and mechanical properties of rectus femoris and biceps femoris using a novel hand-held MyotonPRO device: Relative ratios and reliability. Working Papers in the Health Sciences; Faculty of Health Sciences.

[36] Mencel J., Marusiak J., Jaskólska A., Jaskólski A., Kisiel-Sajewicz K. (2021). Impact of the Location of Myometric Measurement Points on Skeletal Muscle Mechanical Properties Outcomes. Muscle Ligaments Tendons J..

[37] Bravo-Sánchez A., Abián P., Sánchez-Infante J., Ramírez-Delacruz M., Esteban-García P., Jiménez F., Abián-Vicén J. (2022). Five-Compressions Protocol as a Valid Myotonometric Method to Assess the Stiffness of the Lower Limbs: A Brief Report. Int. J. Environ. Res. Public Health.

[38] Hopkins W.G., Marshall S.W., Batterham A.M., Hanin J. (2009). Progressive statistics for studies in sports medicine and exercise science. Med. Sci. Sports Exerc..

[39] O’Brien T.D., Reeves N.D., Baltzopoulos V., Jones D.A., Maganaris C.N. (2010). Muscle-tendon structure and dimensions in adults and children. Am. J. Anat..

[40] Romer C., Czupajllo J., Zessin E., Fischer T., Wolfarth B., Lerchbaumer M.H. (2022). Stiffness of Muscles and Tendons of the Lower Limb of Professional and Semiprofessional Athletes Using Shear Wave Elastography. J. Ultrasound Med..

[41] Wren T.A., Beaupré G.S., Carter D.R. (2000). Tendon and ligament adaptation to exercise, immobilization, and remobilization. J. Rehabil. Res. Dev..

[42] Butler R.J., Crowell H.P., Davis I.M. (2003). Lower extremity stiffness: Implications for performance and injury. Clin. Biomech..

[43] Mersmann F., Bohm S., Arampatzis A. (2017). Imbalances in the Development of Muscle and Tendon as Risk Factor for Tendinopathies in Youth Athletes: A Review of Current Evidence and Concepts of Prevention. Front. Physiol..

[44] Domroes T., Weidlich K., Bohm S., Mersmann F., Arampatzis A. (2025). A Personalized Muscle-Tendon Assessment and Exercise Prescription Concept Reduces Muscle-Tendon Imbalances in Female Adolescent Athletes. Sports Med.-Open.

[45] Brazier J., Maloney S., Bishop C., Read P.J., Turner A.N. (2019). Lower Extremity Stiffness: Considerations for Testing, Performance Enhancement, and Injury Risk. J. Strength Cond. Res..

[46] Xu D., Zhou H., Jie T., Zhou Z., Yuan Y., Jemni M., Quan W., Gao Z., Xiang L., Gusztav F. (2025). Data-driven deep learning for predicting ligament fatigue failure risk mechanisms. Int. J. Mech. Sci..

[47] Jarocka E., Marusiak J., Kumorek M., Jaskólska A., Jaskólski A. (2012). Muscle stiffness at different force levels measured with two myotonometric devices. Physiol. Meas..

[48] Tashiro T., Okugaki T., Abekura T., Kumamoto S., Arima S., Maeda N. (2025). Sex and Side Differences in Muscle Stiffness of Lower Limb Muscles in Healthy Adults. Gait Posture.

[49] McMahon G., Morse C.I., Winwood K., Burden A., Onambélé G.L. (2018). Gender associated muscle-tendon adaptations to resistance training. PLoS ONE.

[50] Khowailed I.A., Lee Y., Lee H. (2022). Assessing the differences in muscle stiffness measured with shear wave elastography and myotonometer during the menstrual cycle in young women. Clin. Physiol. Funct. Imaging.

